# Supplementary material for: Simultaneously Enhanced Thermal Conductivity and Dielectric Breakdown Strength in Sandwich AlN/Epoxy Composites
Source: Nanomaterials (Basel). 2021 Jul 24;11(8):1898. doi: 10.3390/nano11081898 (PMC8401393; doi:10.3390/nano11081898)
Supplement: Supplementary file 1 [file nanomaterials-11-01898-s001.zip › nanomaterials-1309384-supplementary.pdf]

nanomaterials

MDPI

Supplementary Materials

Simultaneously Enhanced Thermal Conductivity and Dielectric Breakdown Strength in Sandwich AlN/Epoxy Composites

Zhengdong Wang [1,2], Xiaozhuo Wang [1], Silong Wang [1], Jieyu He [1], Tong Zhang [1], Juan Wang [1,2] and Guanglei Wu [3,*]

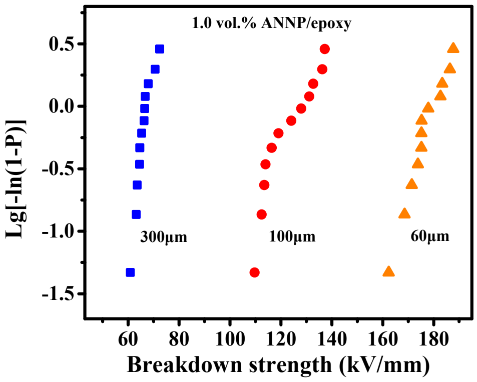

Figure S1. Weibull distribution for breakdown strength of 1.0 vol.% ANNP/epoxy composites with different thickness.

Table S1. Characteristic breakdown strength of ANNP/epoxy composites with different ANNP loading.

Samples	Breakdown strength (kV/mm)
Neat epoxy	64.45
0.3 vol.% ANNP	64.88
0.5 vol.% ANNP	65.62
1.0 vol.% ANNP	66.5
2.0 vol.% ANNP	62.24

Table S2. Thermal conductivity and volume fraction of inner layer and outer layers in sandwich composites.

Sample	Predicted thermal conductivity (W/m·K)					Volume fraction (vol %)		
	K_{c1}	K_{c2}	K_1	K_2	K_3	Φ_1	Φ_2	Φ_3
1.0 vol.% ANNP/epoxy			-	0.200	-			
35.6 vol.% ANMP/epoxy			2.30	-	2.30			
90-120	0.442	1.46				9/30	12/30	9/30
100-100	0.511	1.60				10/30	10/30	10/30
110-80	0.605	1.74				11/30	8/30	11/30
120-60	0.742	1.88				12/30	6/30	12/30
130-40	0.958	2.02				13/30	4/30	13/32
